# Supplementary material for: The interactome of intact mitochondria by cross-linking mass spectrometry provides evidence for coexisting respiratory supercomplexes
Source: Mol Cell Proteomics. 2017 Dec 8;17(2):216–32. doi: 10.1074/mcp.RA117.000470 (PMC5795388; doi:10.1074/mcp.RA117.000470)
Supplement: Supplemental Data [file supp_RA117.000470_133922_0_supp_23978_fzffwf.zip › spectra_annotation/mito_DR_spectra_annotation/374-2-52-1-20-1.pdf]

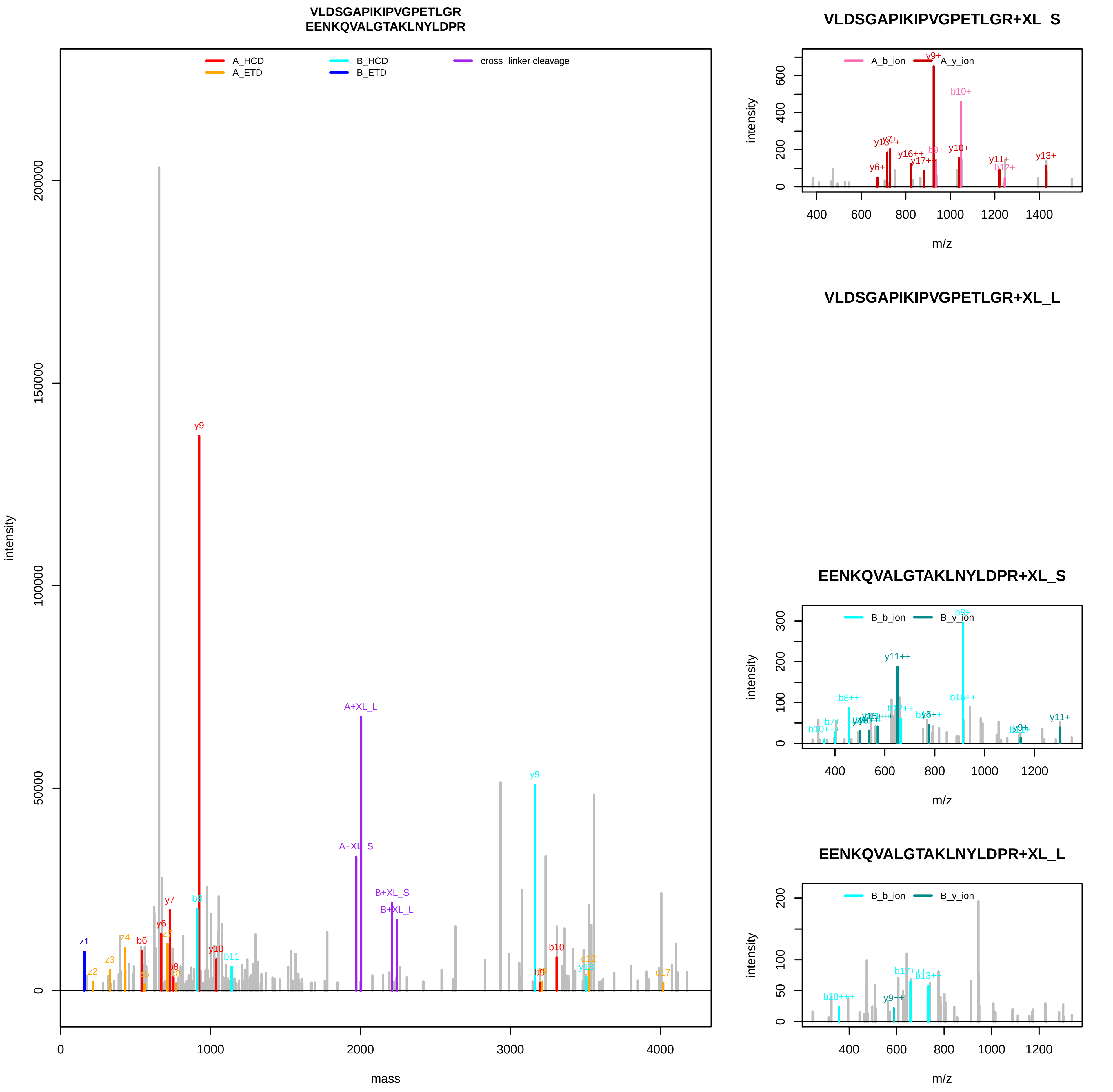

VLDSGAPIKIPVGPETLGR
EENKQVALGTAKLNYLDPR
A_HCD
A_ETD
B_HCD
B_ETD
cross−linker cleavage
intensity
mass
y9
A+XL_L
A+XL_S
B+XL_S
B+XL_L
y9
b10
y7
y6
b8
z1
z2
z3
z4
b6
z7
y10
b11
c17
VLDSGAPIKIPVGPETLGR+XL_S
A_b_ion
A_y_ion
y9+
b10+
y10+
y13+
y11+
b12+
y6+
y16++
y17++
b9+
m/z
VLDSGAPIKIPVGPETLGR+XL_L
EENKQVALGTAKLNYLDPR+XL_S
B_b_ion
B_y_ion
b8+
y11++
b8++
b16++
y11+
y9+
y6+
b12++
b10+++
b7++
EENKQVALGTAKLNYLDPR+XL_L
b17+++
b13++
b10+++
y9++
